# Ovarian Tumor Cured by Galvanization

**Published:** 1878-03

**Authors:** E. Cutter

**Affiliations:** Cambridge, Mass.


### Clinical Reports.

### NOTES FROM PRIVATE PRACTICE.

*A Case of Ovarian Tumor pronounced too feeble for Ovariotomy and reported cured by the Galvanic Current from the Author's Large Battery.*

Certain Chicago surgeons will remember being present at 249 E. Indiana street, June 11, 1877, to witness the operation of galvanization of a fibroid tumor of the uterus. The tumor was multilocular, abdominal and pelvic, evidently connected with the uterus. They will also remember examining the case and concurring in the diagnosis of fibro-cystic disease of the uterus; also, that when the patient was etherized, the writer introduced an electrode into the abdominal tumor. It was evident at once that a mistake had been made, since a microscopical examination revealed the presence of Sluge's compound cell and Drysdale's ovarian cell. The writer stated that he thought it advisable to proceed no further, as the battery, containing nearly thirteen square feet of surface, was inapplicable to this kind of cases.

It was advised to use the writer's small copper and zinc battery on the outside of the abdomen. This has been done, I am informed, and the tumor had not increased at last accounts. It is difficult to retract advice previously given, but in this case it must be done. I was wrong and ought to have proceeded with the operation; that is, if anything is to be learned from the following history:

Early in December last, Dr. Frank K. Paddock, of Pittsfield, Mass., wrote me that Dr. G. A. Pierce, of Lebanon Springs, N. Y., had cured a case of ovarian tumor with my large battery, which belongs to Dr. Paddock, and was loaned to Dr. Pierce for the purpose. This I could hardly believe, as it was entirely contrary to the opinion I had entertained as to the proper use of the battery in question.

Under date of Dec. 27, 1877, Dr. P. wrote substantially as follows: "Miss Norton, aged 34 years, took cold Sept. 1, 1875, while menstruating. She was quite sick at the time, and noticed a bunch over the right ovary. It gradually increased in size. She consulted her brother, a homeœpathic physician, and took medicine without relief. Jan. 2, 1877, she was very large, and Dr. Pierce examined her and pronounced the case ovarian. She was advised to consult Prof. Vandeveer, of Albany, N. Y., an acknowledged authority in these cases. Dr. P. accompanied him, and after a critical examination, Dr. Vandeveer concurred in the diagnosis. On the ground that the patient's health did not admit of ovariotomy, Prof. Vandeveer advised Dr. Pierce to employ the large battery. July 20th, 1877, Dr. Pierce tapped and withdrew 23 lbs. of fluid. Aug. 18th (twenty-nine days afterwards) she was as large, if not larger, than before she was tapped. This day the electrodes of the writer were introduced through the abdominal parietes and the current applied for ten minutes. Six other applications were made up to Nov. 6th, using a progressively longer time, so that for the last three applications the current was continued for half an hour. There was a marked diminution of the fluid after each application, and on Nov. 6th there was no more fluid in the sac. Dec. 3d, Prof. Vandeveer examined her at Albany and found her cured.

Jan. 10th, 1878, Dr. P. writes: "The fluid gave the characteristic test for fat and albumen. It contained the so-called ovarian cell, neutral reaction; sp. gr. 1020." Dr. Pierce adds, "No sign of filling as yet." In a private note Prof. Vandeveer confirms the above, and says there is no doubt of the ovarian character of the sac, and of the cure."

REMARKS.—This case should not be relied on until it has stood the test of time. Prof. Vandeveer writes that he will report the case in full, and for this report we shall look with interest. This abstract is made to correct the advice formerly given in a similar case.

E. CUTTER, M. D., Cambridge, Mass.

